# Behavioral phenotyping identifies autism-like repetitive stereotypies in a Tsc2 haploinsufficient rat model

**DOI:** 10.1186/s12993-025-00284-z

**Published:** 2025-07-03

**Authors:** Antonia Ramme, Mirjam Zachow, Bettina Habelt, Iveta Vojtechova, Tomas Petrasek, Robert Waltereit, Nadine Bernhardt

**Affiliations:** 1 Department of Psychiatry and Psychotherapy, University Hospital Carl Gustav Carus, Technische Universität Dresden, Dresden, Germany; 2 National Institute of Mental Health, Klecany, Czechia; 3 Department of Child and Adolescent Psychiatry, University Medical Center, Göttingen, Germany; 4 Ruhr University Bochum, Bochum, Germany

**Keywords:** Autism spectrum disorder, Tuberous sclerosis complex, Repetitive behavior, Eker rat

## Abstract

Besides deficits in social communication and interaction, repetitive behavior patterns are core manifestations of autism spectrum disorder (ASD). Phenotypes are heterogeneous and can range from simple lower-order motor stereotypies to more complex higher-order cognitive inflexibility and fixated interests. Due to ASD’s multifaceted etiology, animal models are often generated from monogenic diseases associated with ASD, such as Tuberous Sclerosis Complex (TSC), and are expected to copy behavioral core deficits to increase the model´s translational value for ASD disease research and novel treatment development. The global haploinsufficient *Tsc2*^*+/−*^ Eker rat model has been shown to display ASD core symptoms in the social domain. However, the presence and extent of aberrant repetitive behavior patterns in the Eker rat remain to be investigated. Thus, the present study applied a set of behavioral tests to determine the repetitive behavioral profile in *Tsc2*^*+/−*^ Eker rats and used brain-region-specific neurotransmitter analysis to support findings on a molecular level. *Tsc2*^*+/−*^ animals demonstrated lower-order repetitive behavior in the form of excessive self-grooming and nestlet shredding under non-stressful conditions that co-occurred alongside social interaction deficits. However, no higher-order repetitive behavior was detected in *Tsc2*^*+/−*^ rats. Interestingly, *Tsc2*^*+/−*^ rats exhibited increased levels of homeostatic dopamine in the prefrontal cortex, supporting the link between aberrant cortical dopaminergic transmission and the appearance of lower-order repetitive phenotypes. Together, our results support the *Tsc2*^*+/−*^ Eker rat as a model of ASD-like behavior for further investigation of ASD-related development and neurobiology.

## Background

Autism Spectrum Disorder (ASD) is a neurodevelopmental disorder defined by deficits in two core behavioral domains: social interaction and repetitive behavior patterns [1, 2]. Despite a high global prevalence of approximately 1% [3], efficacious drugs are missing, and early behavioral interventions remain the most promising treatment option [4]. The biggest impeding factor for drug development remains ASD´s complex and incompletely understood etiology. Disease pathogenesis arises from a multifaceted interaction between a range of genetic and environmental factors that together give rise to a wide symptom heterogeneity across ASD patients. Moreover, ASD is linked to over 100 candidate genes and a myriad of genetic risk factors like chromosomal rearrangements and copy number variations, with mainly small to moderate effect sizes [5–9], thus posing an additional challenge to generate disease models with high translational accuracy.

Similarly, different transgenic ASD rodent models exhibit unique sets of behavioral alterations that fit the high aetiologic and symptomatic heterogeneity in humans [10–12]. While social behavior is well-described in most ASD rodent models, characterizations of repetitive behavior profiles are less commonly reported. Repetitive behaviors in ASD patients include a range of motor patterns divided into (a) “lower-order” (stereotyped or repetitive motor movements), (b) more complex “higher-order” responses (compulsions, insistence on sameness, cognitive inflexibility and adherence to routine) and (c) highly restricted interests such as unusual object fixation [1, 13]. In rodent ASD models, strain-specific repetitive patterns can be revealed by applying combinations of behavioral tests that cover multiple response categories.

Commonly used models are often based on single-gene disorders associated with ASD, such as Tuberous Sclerosis (TSC) and Fragile X syndrome [14, 15], or high-effect risk genes like *SHANK3*, *NLGN3*, and *NLGN4*, as they offer high construct validity and thereby increase relevance [12, 16–18]. Among genetic aberrations reported in ASD, the *TSC2* gene remains one of the major contributors [19]. TSC is an early-onset, monogenic disorder caused by loss-of-function mutations in the *Tsc1* or *Tsc2* gene. Patients present with autism-like neurobehavioral phenotypes, including social deficits and epileptic seizures [20–22], and are co-diagnosed with ASD in up to 69% of cases, suggesting genetic linkage of ASD and TSC [19, 23]. While the connection between genetic and anatomical alterations is not yet fully understood, molecularly, *Tsc1* and *Tsc2* mutations are known to result in overactive mTORC1 (mammalian target of rapamycin complex 1), thereby interfering with metabolic processes including cell growth, proliferation, differentiation, and protein synthesis [24]. Hyperactive mTOR signaling is associated with impaired neurodevelopment, synaptic plasticity, and signaling, and thus strongly correlates with neurodevelopmental diseases like epilepsy and ASD [25, 26].

The *Tsc2*^*+/−*^ Eker rat model carries a spontaneous germ-line mutation of the *Tsc2* gene [27, 28], making it a valuable tool for studying TSC etiology and its implications in ASD [21, 29–31]. Reportedly, Eker rats recapitulate brain abnormalities and cellular pathology present in human TSC [21, 32–34], show altered synaptic plasticity [33], and present with mild but consistent autism-like social impairments [35–37]. The observed social deficits were found to improve upon selective inhibition of mTORC1 in *Tsc2*^*+/−*^ animals, supporting hyperactive mTORC1 signaling as a potential driver of behavioral manifestations in ASD [37]. However, along with concerns about absent spontaneous seizure activity, the lack of repetitive behavior characterization sparks debate about the suitability of the Eker rat as an ASD model [21].

Therefore, the present study aimed to provide an extended characterization of ASD-like repetitive behavior in Tsc2+/- Eker rats by exposing animals to a test battery addressing both lower- and higher-order repetitive phenotypes to strengthen the model’s translational value in ASD research.

## Methods

### Animals

All experiments were performed in the heterozygous Eker rat strain with *Tsc2*^+/−^ mutation on a Long-Evans background, RRID: RGD_625624, and their wild-type (wt, *Tsc2*^*+/+*^) littermates, which were used as controls. Animals were bred at and obtained from the National Institute of Mental Health in Klecany, Czechia, at 8 weeks of age. Acclimatization and daily handling were performed for 4 weeks prior to behavioral experiments. The animals were housed in pairs of 2–3 in standard housing cages (Makrolon^®^, Type IV-S, Tecniplast Deutschland GmbH, Hohenpeißenberg, Germany) in a controlled environment of 21–24 °C and an average humidity of 55% under an automated 12 h/12 h day-night cycle. Food and water were provided *ad libitum*. Experiments took place during the late-day phase, and animals’ weight was controlled twice a week.

### Behavioral testing

**Fig. 1:**
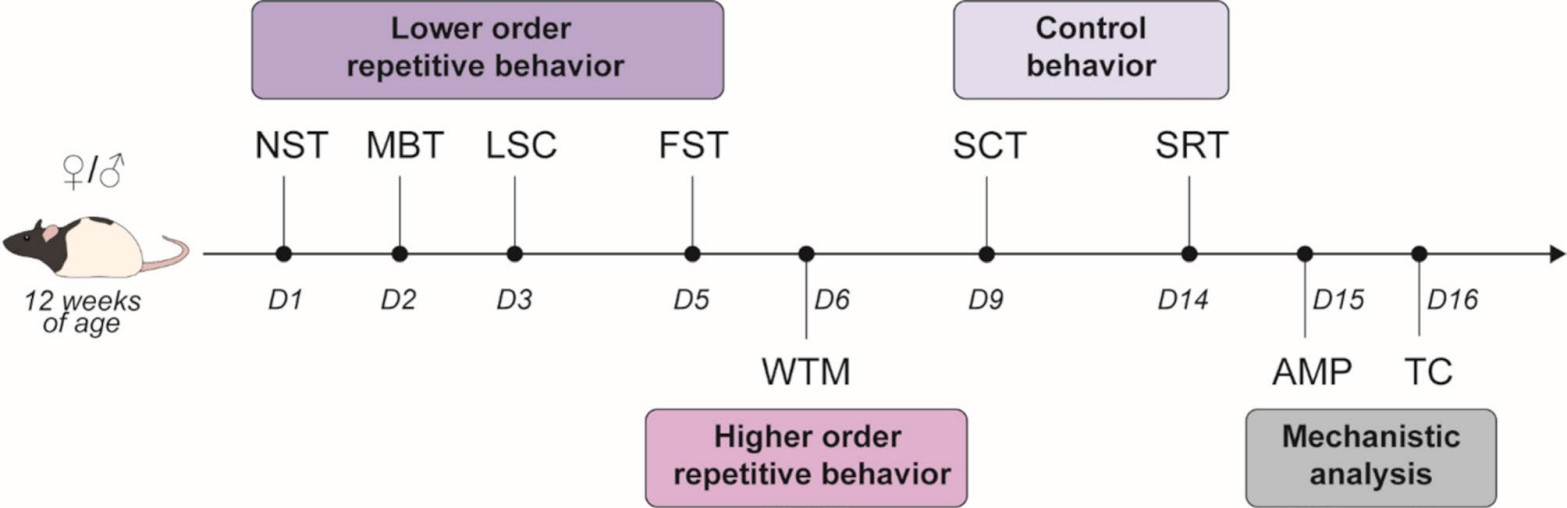
Behavioral testing sequence. All *Tsc2*^*+/−*^ and wt rats were subjected to a sequential behavioral test battery including the nestlet shredding test (NST), marble burying test (MBT), light-sound-confinement test (LSC) and forced swim test (FST) to reveal lower-order repetitive behavior and the water-T-maze test (WTM) to test for higher-order repetitive stereotypies. The sucrose consumption test (SCT) and social recognition test (SRT) were performed to exclude that anhedonic tendencies influence obtained results and confirm the context of social impairment in our model (control). Finally, an amphetamine-induced stereotypic behavior test (AMP) was performed, followed by tissue collection (TC) for post-mortem analysis. Behavioral testing started when animals of both sexes were 12 weeks of age

Behavioral experiments started when animals were 12 weeks of age, in accordance with relevant literature on social behavior of *Tsc2*^*+/−*^ Eker rats [35]. A total of 11 *Tsc2*^*+/−*^ and 11 wt rats were used and subjected to the behavioral testing sequence and following post-mortem analysis. Sample size was calculated a priori using G*Power. Genotype groups included both male (*n* = 6) and female (*n* = 5) animals, respectively. Experiments were conducted in two separate birth cohorts, with sex being counterbalanced across cohorts. Experimental testing and analysis were performed by trained and blinded experimenters. A broad behavioral test battery assessing both lower- (marble burying test, nestlet shredding test, light-sound-confinement test, forced swim test) and higher-order (water-T-maze test) repetitive behavior was used. Additional readouts for social behavior (social recognition test), reward function (sucrose consumption test), and cognition (water-T-maze test) were collected to report repetitive behavior alterations in the context of already known abnormalities. Behavioral tests were performed in a fixed sequence depicted in Fig. 1, allowing for a minimum of 24 h resting period between tests to limit stress and potential carryover effects, however, interferences with following tests cannot be fully excluded.

#### Nestlet shredding test

The nestlet shredding test was used to detect lower-order repetitive behavior in non-stressful conditions [38]. Animals were placed individually in a standard cage containing bedding material, fresh nestlet material, and a square tissue paper (5 cm × 5 cm, weighing 2.5 g) in the opposite corner. The test was conducted for 30 min without food or water supply before the animals were returned to their home cage. The remaining unshredded nestlet material was then weighed [38, 39].

#### Marble burying test

The marble burying test was used to detect lower-order motor stereotypy in the form of repetitive digging behavior in non-stressful conditions [38, 40]. Animals were placed individually in a standard cage with bedding material filled up to 5 cm height, in which 9 clean, commercially available glass marbles (approx. 15 mm diameter) were evenly distributed on half of the cage´s surface. Animals were allowed to explore for 30 min without food and water supply, after which they returned to their home cage, and the number of buried marbles that were at least 2/3 covered in bedding material was counted [38, 39].

#### Light-sound-confinement test

A light-sound-confinement test (LSC) was performed to assess lower-order repetitive behavior to acute increasingly stressful conditions through the application of combined auditory and visual stress in a new confining environment. The animals were placed in a plexiglass tube (30 cm height and 12 cm diameter) (Stoeltus Co., Ireland) fixed on a standard cage and the tube was fitted with a commercially available loudspeaker (SoundCore Select 2 Bluetooth Loudspeaker, Anker, Germany) and a light source (KL 1600 LED, Schott AG, Germany). Behavior of the rats was filmed from two sides using ManyCam (Visicom Media Inc.) for 30 min in total. For the first 10 min, activity of the animals was recorded without audiogenic or visual stressors, reflecting a mild stress condition applied through the new and spatially limited arena (pre-test phase). During the following 10 min test phase, animals were exposed to both visual and auditory stressors. Light was applied at a maximum of 1400 lx combined with white noise bursts (100 dB SPL, 50 ms) (programmed with Audacity Version 2.3.0 and Matlab Version R2019a), played with random interstimulus intervals ranging between 10 and 20 s. After removal of both stressors, the activity of the animals was recorded for another 10 min (post-test phase). Total grooming and digging time were analyzed for each phase [41–43].

#### Forced swim test

The forced swim test (FST) was conducted to measure active coping strategies in stress response [44]. Further, response behavior was assessed for repetitive patterns. For habituation, animals were placed in a glass cylinder (60 cm height, 24 cm diameter) filled with 23 ± 1 °C water to 30 cm height for 15 min individually. Afterward, animals were dried under a red-light source (SIL-6 red light lamp Beurer, Hans Dinslage GmbH, Germany) and placed back in their home cage. After 24 h, animals were tested in a water height of 40 cm, and activity was recorded for 5 min. The total time of immobility per animal was determined using the software EthoVision^®^ XT (Noldus Information Technology, Netherlands) [45, 46]. Additionally, potential repetitive climbing patterns were manually assessed by a trained experimenter, and potential uninterrupted repeated circular swimming was evaluated in EthoVision´s heat map feature.

#### Water-T-maze

The water-T-maze (WTM) test was conducted to detect higher-order repetitive behavior as well as assess learning and memory [47]. The experiment was conducted in a T-shaped pool (Maze Engineers, Conduct Science©, USA) filled with 23 ± 1 °C opaque water (XSL titanium white, Kremer pigments, Germany), which consisted of a long stem (L 70 cm, W 15.5 cm, H 46 cm) that bifurcates into two arms (L 121 cm × W 15.5 cm × H 46 cm). A transparent platform (15.5 cm x 15.5 cm, 24 cm height) was randomly placed in one of the two arms of the T-maze and was submerged 1 cm below water level. On day one (acquisition phase), animals were trained to find the platform position in a fixed arm. Animals were placed in the starting arm and allowed to choose either direction after reaching the end of the stem. Once an animal reached either arm within a test period of 60 s, the arm was closed with a plexiglass door (47 cm × 20 cm), confining the animal for 5 s before removal to a holding cage for 10 s and subsequent test repetition. The training was repeated until the animal chose the correct arm and escaped onto the platform in five consecutive trials. On day two, 24 h later, animals were retrained by repeating the previous day´s test procedure (repetition phase). Once position learning was completed, the platform was switched to the opposite arm of the T-maze, and the test procedure was repeated as described (reversal test). During each holding period outside of the maze, animals were dried under a red-light source (SIL-6 red light lamp Beurer, Hans Dinslage GmbH, Germany). The number of attempts required by each animal to make five consecutive correct decisions was recorded; the maximum number of trials was limited to 25 per day.

#### Sucrose consumption test

The sucrose consumption test used to assess reward function and depressive episodes [48], served to evaluate anhedonic tendencies linked to repetitive behavior. Animals were habituated to a standard bottle of sweetened condensed milk (Nestlé, Milchmädchen, (1:3)) in their home cage for 30 min. After 24 h, animals were habituated to the single housing standard cage type III (Makrolon^®^, Tecniplast Deutschland GmbH, Germany) for 30 min and subsequently food restricted for 21 h (15 g per animal). On the day of testing, animals were exposed to the bottle containing sucrose solution for 10 min. Bottle weight was recorded before and after the test phase. Animals were weighed on each test day as well as the day after sucrose consumption [49].

#### Social recognition test

The social recognition test was used to evaluate sociability towards an unknown partner rat. Animals were kept in isolation for three consecutive days and were familiarized with the test box (45 cm × 45 cm, LE802S Panlab square arena, S.L.U. Panlab, Spain) for 1 h every day. On the test day, each experimental animal was presented with an unfamiliar sex- and age-matched conspecific of the same strain and placed in a locked startle box (Grid Rod Animal Holder, OCB Systems Ltd., United Kingdom) with openings for nose-to-nose contact. Experimental animals were allowed to explore or interact for a test period of 60 s in a total of four trials with the same social-partner animal in intervals of 10 min. In a fifth trial, experimental animals were presented with a novel social partner animal. Following each trial, the social-partner animal was returned to their home cage for the 10 min interval time, and the startle box was disinfected. All trials were videotaped, and the examination time spent in close proximity was manually determined [50].

#### Amphetamine-induced stereotypic behavior test

An amphetamine-induction test was conducted to characterize the effect of amphetamine on repetitive behavior stereotypies. Animals were injected intraperitoneally with 2.0 mg amphetamine (Lipomed AG, Switzerland) per kg body weight, based on the emergence of amphetamine-induced stereotypy in wt animals above doses of 2 mg/kg [49]. Rats were placed in a testing box (46.5 × 46.5 cm × 44 cm) and video-recorded for 2 h. Behavior following amphetamine application was analyzed in 5 min intervals, and the most prominent behavior was scored for each interval. Scoring was performed based on an adapted protocol described in Kelly et al. (1975) [51] by dividing behavior in (0) no locomotor activity (sleeping, residing without apparent sniffing) (1) limited exploratory activity (discontinuous sniffing, rearing or grooming) (2) locomotion (frequent rearing, sniffing) and (3) stereotypic behavior (repetitive grooming, rearing and licking). Scoring was performed manually by a single trained and blinded experimenter. Blinding was achieved by number-coding animals for video-analysis to eliminate genotype-bias.

### Post-mortem high-performance liquid chromatography

Animals were intraperitoneally anesthetized using pentobarbital (60 mg/kg) and decapitated. Whole brains were extracted and frozen in methylbutane for 2 min at -20 to -40 °C and stored at -80 °C. Bilateral micro-punches (Ø 1 mm) of prefrontal cortex (PFC) and striatum (caudate putamen, CPu) tissue were homogenized in 500 µl 0.1 M perchloric acid by ultrasonication that was applied for 3 × 10 s on ice. After protein quantification (PierceTM 660 nm Protein Assay; Thermo Fisher Scientific Inc., USA), homogenates were centrifuged for 15 min at 13,000 g at 4 °C. The supernatant was separated via HPLC (1260 Infinity II LC System, OpenLab LC ChemStation Software, Agilent Technologies, USA) on a PRONTOSIL 120-5-C18SH (VDS Optilab, Germany) analytical column, followed by electrochemical detection (Coulochem III, Thermo Fisher Scientific Inc., USA) of dopamine (DA), 3,4-dihydroxyphenylacetic acid (DOPAC), and homovanillic acid (HVA) levels as µg/g protein. Dopamine turnover was calculated from concentration levels, adjusted to the protein content of the sample, as neurotransmitter to metabolite ratio (DA/(DOPAC + HVA)).

### Statistical analysis

Statistical analysis was performed using GraphPad Prism 9.5.1 and Jamovi 2.6.44. All behavioral tests as well as HPLC measures were analyzed using a two-way analysis of variance (ANOVA) for the main effect of genotype (wt, *Tsc2*^+/−^ Eker) and sex (male, female). In consideration of our small dataset, we did not test for normality but relied on the visual inspection of qq-plots for residuals and the robustness of ANOVA, to violations of normality, especially when the sample sizes are small and equal across groups [52]. Tukey’s HSD was used to adjust for multiple post hoc comparisons. Correlation analysis was performed for findings with a significant genotype effect using Spearman´s correlation coefficient. No animals were excluded from analysis. Statistical significance was set at *p* < 0.05.

## Results

### *Tsc2* haploinsufficiency increases lower-order repetitive behavior patterns

To determine if the *Tsc2*^*+/−*^ genotype induces ASD-associated behavior in rats, a set of behavioral tests was performed on the Eker model to reveal repetitive behavior patterns.

**Fig. 2:**
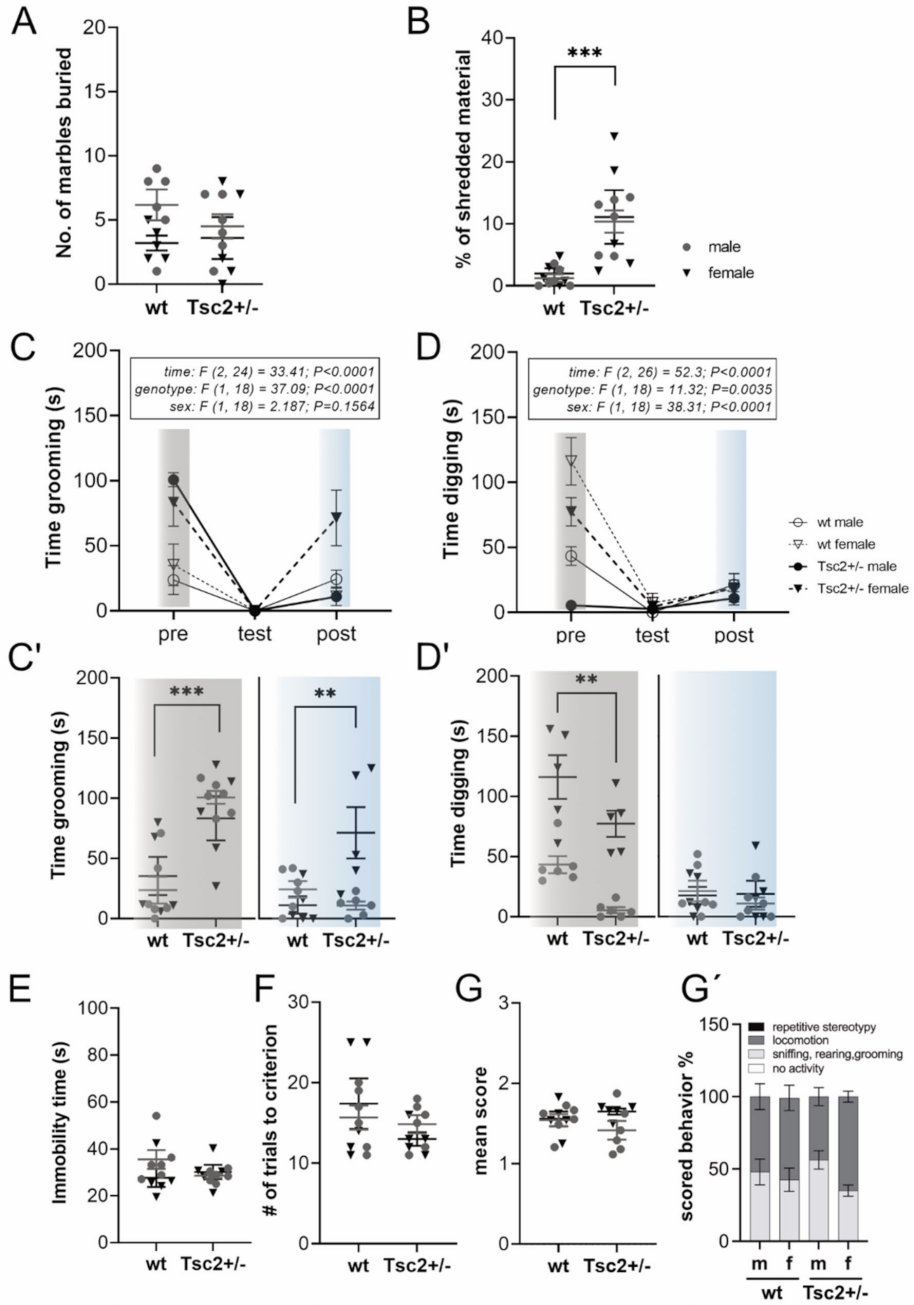
*Tsc2*^*+/−*^Eker rats display increased lower-order repetitive behavior without higher-order repetitive abnormalities. **A** In the marble burying test, no significant change in the number of marbles buried was observed between genotypes (*p* = 0.589) or sex (*p* = 0.382). **B** In the nestlet shredding test, *Tsc2*^+/−^ rats showed significantly increased shredding behavior compared to wt (*p* = 0.001). **C**,** D**,** C’**,** D’** Data obtained from a light-sound confinement test. During the pre-test habituation, *Tsc2*^*+/−*^ rats showed significantly increased grooming (*p* = 0.001). In case of digging, a significant sex effect was observed, with females displaying more digging behavior than males (*p* = 0.001). Further, a significant effect of genotype was revealed (*p* = 0.002). In the test phase, both genotypes exhibited minimal digging and grooming behavior. During post-test recovery, a significant interaction between sex and genotype was observed in case of grooming (*p* = 0.004). *Tsc2*^*+/−*^ female rats showed increased grooming behavior compared to wt and *Tsc2*^*+/−*^ males. Genotype and sex did not affect digging behavior in the post-test phase (*p* = 0.578). C´ and D´ show pre- and post-test phase with individual values. **E** In the FST, no significant effect of genotype (*p* = 0.497) and sex (*p* < 0.961) was observed. **F** No effect of genotype (*p* = 0.163) or sex (*p* = 0.978) was detected in the reversal learning paradigm of the WTM test. Depicted are the total number of trials needed on day 2. **G**,** G´** After systemic administration of amphetamine, *Tsc2*^*+/−*^ animals did not show increased susceptibility to repetitive behavior induction (*p* = 0.846). **G** Depicted is the mean score of behavior categories following classification as described in the methods section. **G´** Shown are the proportions of scoring classifications per genotype. Animals of all genotypes (male (m), female (f)) showed predominant behavior of only two categories: (1) limited exploratory activity (sniffing, rearing, grooming) and (2) locomotion, but no resting (0) or stereotypic behavior (3)

First, lower-order restricted behavior was observed under non-stressful conditions using the nestlet shredding and marble burying test. Both behavioral tests are commonly used to reveal stereotypic repetitive behavior [38, 40]. While no differences in marble-burying behavior were observed between wild-type (wt) and *Tsc2*^*+/−*^ animals (Fig. 2A), *Tsc2*^*+/−*^ rats displayed significantly increased nestlet shredding behavior (*F*(1, 18) = 16.39, *p* = 0.001) (Fig. 2B), indicating lower-order repetitive behavior in Eker rats.

Since repetitive behavior represents a strategy to deal with stress and anxiety, next, stereotyped movements were assessed under stressful conditions. The Light-Sound-Confinement test was utilized to investigate repetitive digging and grooming behavior under the application of multiple stressors. First, animals were subjected to the pre-test phase, in which they adjusted to the novel test environment in the absence of additional stressors. *Tsc2*^*+/−*^ rats showed significantly increased grooming behavior compared to wt (Fig. 2C, C´; *F*(1, 18) = 23.47, *p* = 0.001), further suggesting repetitive behavioral abnormalities in the Eker rat model. Conversely, digging time was significantly reduced in *Tsc2*^*+/−*^ rats compared to wt in the pre-test phase (Fig. 2D, D´; *F*(1, 18) = 13.43, *p* = 0.002), but did not significantly correlate with grooming behavior in the same test. Notably, a significant sex effect was observed in the case of digging in the pre-test phase (Fig. 2D, D´; *F*(1, 18) = 47.92, *p* = 0.001), as female rats displayed higher levels of digging behavior than males. Interestingly, while repetitive nestlet shredding exhibited a negative correlation with digging in the pre-LSC (*r*_*s*_(19) = -0.474, *p* = 0.03), it positively correlated with repetitive pre-LSC grooming (*r*_*s*_(19) = 0.531, *p* = 0.01), suggesting that both behaviors reflect a similar lower-order stereotypy pathology.

Next, animals were subjected to the test phase, where unpredictable visual and acoustic stressors were introduced to elevate stress levels. Neither genotype demonstrated grooming behavior nor digging behavior during stressor application (Fig. 2C, D). Interestingly, a sex and genotype interaction for grooming was observed during recovery in the post-test phase as female *Tsc2*^*+/−*^ rats displayed significantly increased grooming behavior compared to male *Tsc2*^*+/−*^ and wt animals (Fig. 2C, C´; *F*(1, 18) = 11.22, *p* = 0.004). In contrast, digging behavior did not significantly differ between genotypes or sex during recovery in the post-test phase (Fig. 2D, D´). However, animals might need more time to recover from the stress phase, thus, the occurrence of a genotype effect at later timepoints cannot be excluded.

In conclusion, while *Tsc2*^*+/−*^ animals were found to display lower-order repetitive behavior in the form of increased grooming in non-stressful conditions, high-stress application in the Light-Sound-Confinement (LSC) test did not exacerbate repetitive behavior patterns in the Eker rat. However, female *Tsc2*^*+/−*^ rats might recover faster following high-stress levels and showed higher compulsive grooming behavior compared to wt. No repetitive digging behavior was found in the Eker rat model.

To test whether *Tsc2*^*+/−*^ rats show altered mobility as a stress response, animals were subjected to the forced swim test (FST). The total time of immobility did not differ significantly between *Tsc2*^*+/−*^ and wt animals (Fig. 2E), suggesting that stress-coping responses involving mobility are not altered in the Eker rat model. Additionally, no repetitive behavior that could present as repetitive circling or climbing was detected in *Tsc2*^*+/−*^ rats.

**Fig. 3:**
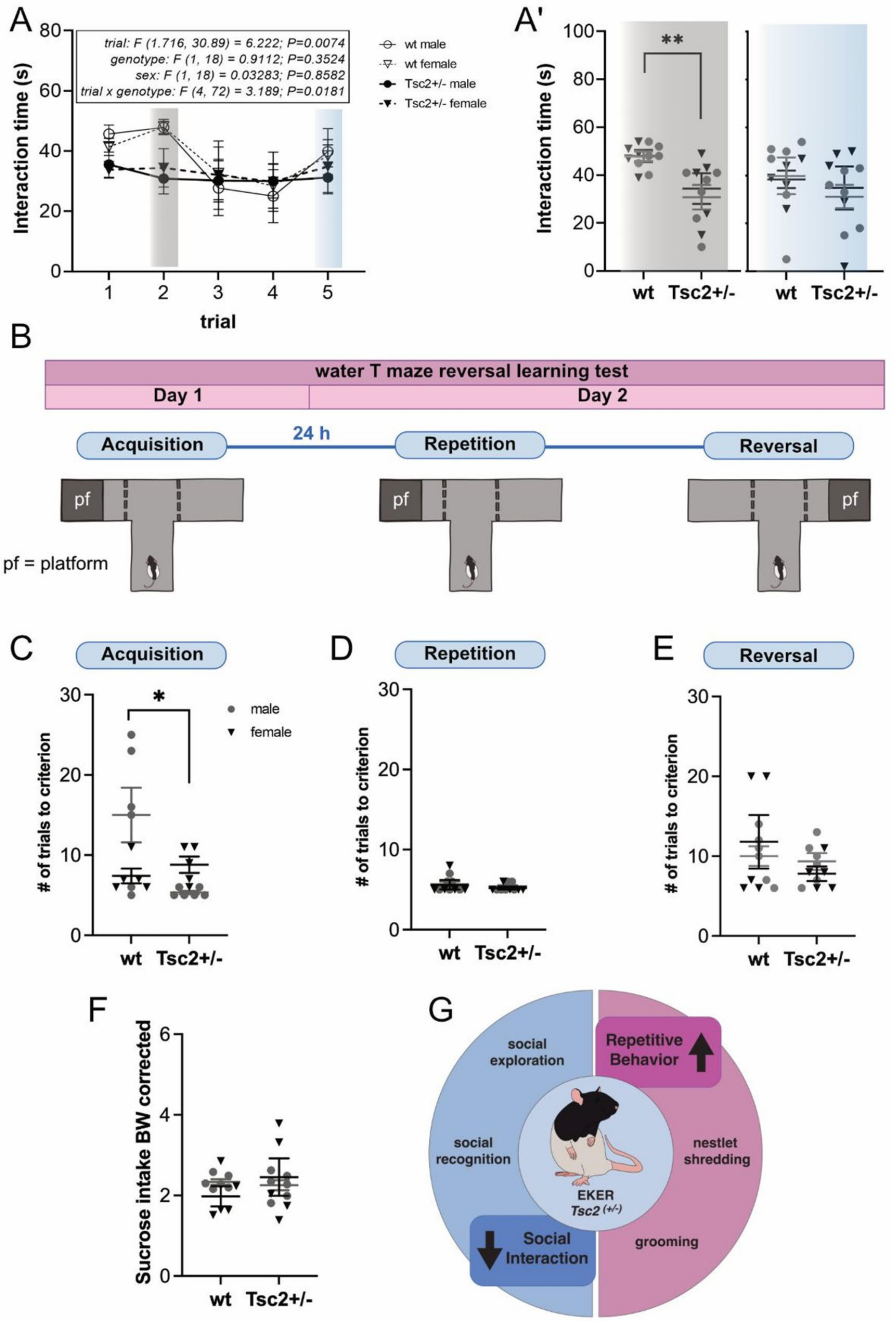
Eker rats present with changes in social interaction. **A** No significant genotype effect was observed in the social recognition test (*p* = 0.353). However, *Tsc2*^+/−^ rats displayed no habituation in subsequent sessions and no enhancement of exploration upon novel social stimulation. **A´** Comparison of social interaction in trial 2 and 5. In trial 2, a significant effect of genotype was observed (*p* = 0.003) with *Tsc2*^*+/−*^ showing decreased social interaction time compared to wt. **B** Experimental scheme of the water T-Maze test. **C-E** T-Maze test performance revealed no genotype effect on spatial memory in initial learning (acquisition phase, C, *p* = 0.052), repetition phase (D, *p* = 0.321), and reversal test (E, *p* = 0.163). However, in the acquisition phase, a significant interaction of sex and genotype was observed (*p* = 0.012), as male *Tsc2*^*+/−*^ rats required significantly fewer trials than male wt rats (*p* = 0.001). **F** In the sucrose consumption test, no significant effect of genotype was detected (*p* = 0.441). **G** Graphical summary of altered ASD core domains in the Eker rat model

The presence of higher-order repetitive behavior was investigated using the water T-maze test (WTM), as its reversal learning paradigm reveals an animal´s ability to switch behavioral strategies. Higher-order repetitive behavior would have presented as perseverative behavior by not successfully completing the task criterion, a consistent choice of novel platform location; thus, animals would reach the maximum number of trials on day 2 of the WTM. Instead, both wt and *Tsc2*^*+/−*^ rats completed the task successfully with no significant difference (Fig. 2F). Both recalling of position habit learning (repetition phase) and finding an unknown platform position (reversal phase) showed no significant difference in attempts needed to fulfill criterion (Fig. 3D, E). Thus, unaltered performance in the WTM suggests that the Eker rat does not demonstrate a significant lack of behavioral response variability and therefore does not display higher-order repetitive behavior. 

Stress-induced repetitive behavior is thought to act as anxiety relief by activating the dopaminergic reward system, thereby representing an important strategy for emotional regulation in ASD and obsessive-compulsive disorder models [53]. In fact, abnormal lower-order repetitive stereotypies are associated with overactivation of the dopaminergic cortical basal ganglia circuitry [54]. Accordingly, treatment with the indirect dopamine agonist amphetamine exacerbates repetitive motor stereotypies and can reveal underlying changes in the dopamine (DA) system in rodent models [55]. Therefore, we opted to investigate whether administering amphetamine at a dosage of 2 mg/kg would reveal signs of repetitive behavior in Eker rats. To determine if the Eker rat model presents with altered DA function and connected occurrence of repetitive behavior, an amphetamine challenge protocol was applied. Behavioral categorization using an adapted scoring protocol from Kelly et al. (1975) [51] revealed that both wt and *Tsc2*^*+/−*^ animals mainly presented with limited (1) and frequent exploratory behavior (2) (rearing, sniffing, grooming) but did not exhibit repetitive stereotypic behavior (3) (repetitive rearing and grooming) (Fig. 2G). A mean behavioral categorization score was calculated to show the overall locomotive activity of sex and genotype. Repeated ANOVA measures did not reveal significant effects of sex on behavior categories, so data from males and females were combined. No significant effect on the extent and type of locomotion was found between wt and *Tsc2*^*+/−*^ animals after amphetamine injection (Fig. 2G, G´). Thus, the application of amphetamine provoked unaltered responses in *Tsc2*^*+/−*^ animals and did not induce repetitive behavior.

In conclusion, the Eker rat model was found to display lower-order repetitive behavior alterations under low-stress conditions in the form of excessive nestlet shredding and grooming. Surprisingly, the application of stressors did not exacerbate repetitive behavior patterns. Furthermore, no higher-order repetitive behavior was detected, indicating sufficient behavioral flexibility in *Tsc2*^*+/−*^ rats. Lastly, the Eker model seems to display wt-like behavioral responses following manipulation of the dopamine system through amphetamine administration, suggesting no major alterations of the DA system to be present.

### Repetitive behavior alterations in the Eker rat are observed in a context of impaired social behavior

Apart from repetitive stereotypies, ASD presents with deficits in social behavior and communication, as well as cognitive impairment and behavioral inflexibility [1]. While autistic-like social behavior has been reported in Eker rats, learning and memory performance was found to be unaltered, suggesting normal cognitive function [35].

To validate that *Tsc2*^*+/−*^ rats show previously reported deficits in social interaction, a social recognition test was performed. First, rats were exposed to an unfamiliar rat. Eker rats showed overall less interaction duration compared to wt during three out of five trials (Fig. 3A), with a significantly decreased interaction time during the second trial (Fig. 3A´, *F*(1, 18) = 12.47, *p* = 0.003), confirming the previously reported general decrease in social interest. Wt animals expectedly displayed variety in social exploratory behavior, with a gradual decrease in interaction during the first four trials and an increase in interaction upon the introduction of a novel actor rat in trial 5, indicating normal social recognition. In contrast, *Tsc2*^*+/−*^ animals were found to maintain similar levels of interaction time across all trials, independent of actor rat familiarity or novelty (Fig. 3A), suggesting restricted social interest and alterations in social recognition in Eker rats.

*Tsc2* haploinsufficiency was found to cause changes in activity-dependent hippocampal synaptic plasticity [33]. To assess whether Eker rats show signs of impaired spatial memory, which is dependent on hippocampal learning, animals were challenged with the water T maze test (Fig. 3B-E). During the acquisition phase, a significant interaction of sex and genotype was observed (*F*(1, 18) = 4.353, *p* = 0.012) as male *Tsc2*^*+/−*^ rats needed significantly fewer trials than wt males to learn the correct arm position (Fig. 3C; *p* = 0.001). However, no negative effect of the *Tsc2*^*+/*^ genotype on learning speed was observed, suggesting that learning is not impaired in Eker rats. In the repetition test, 24 h later, wt and *Tsc2*^*+/−*^ animals found the correct arm position equally well (Fig. 3D). Similarly, no significant differences in number of trials needed to criterion were detected between genotypes in the reversal test (Fig. 3E). These results indicate that spatial memory formation is not affected in Eker rats and are in line with previous reports [35].

To test if alterations in reward function and motivation might influence behavioral test results, a sucrose consumption test was performed. No difference between genotypes nor interaction effects have been observed (Fig. 3F), indicating that *Tsc2*^*+/−*^ animals show intact reward function and do not develop anhedonic tendencies.

In summary, Eker rats show signs of social behavior impairment in the form of abnormal social interest. On the contrary, spatial learning and memory, as well as reward function, remained unaffected by *Tsc2* haploinsufficiency. Herein, we provide evidence that the *Tsc2*^*+/−*^ animal model of ASD presents with an overt repetitive behavior pattern that occurs alongside social behavior impairments (Fig. 3G, G´).

### Eker rats show evidence for impaired PFC-dependent dopaminergic signaling

**Fig. 4:**
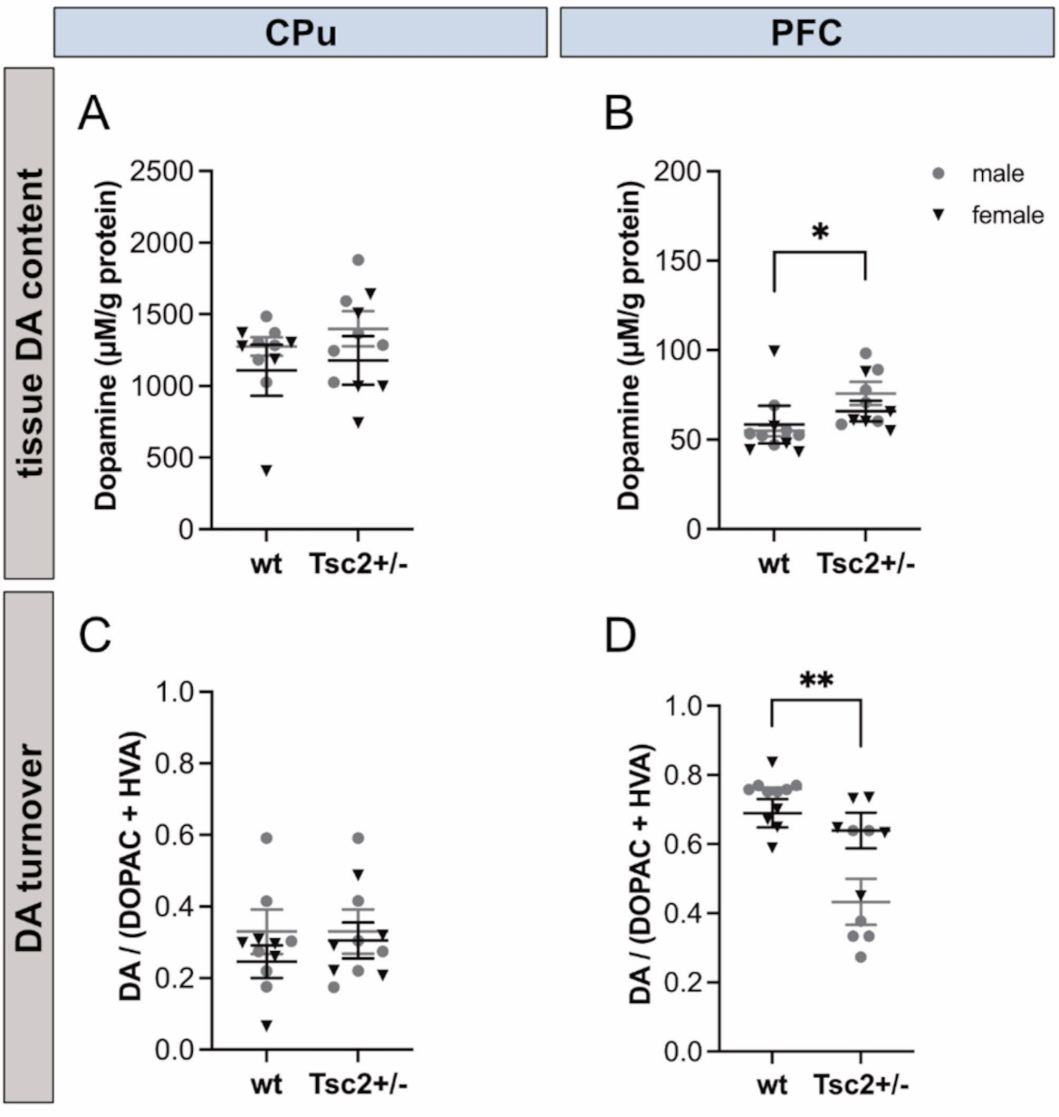
Eker rats show altered dopamine metabolism in the PFC. **A**,** B** HPLC analysis of dopamine (DA) levels in both striatum (CPu) and PFC. **A** DA levels in the CPu were not significantly altered in *Tsc2*^+/−^ rats compared to wt (*p* = 0.484). **B ***Tsc2*^+/−^ rat PFC tissue showed significantly increased levels of DA (*p* = 0.049). **C**,** D** DA turnover based on HPLC analysis of dopamine metabolites DOPAC and HVA in both CPu and PFC. **C** DA turnover in the CPU was not significantly altered in *Tsc2*^+/−^ rats compared to wt (*p* = 0.61). **D** In PFC tissue a significant interaction of sex and genotype was observed, as male *Tsc2*^+/−^ rats showed significantly increased DA turnover compared to wt and female *Tsc2*^+/−^ animals (*p* = 0.01). No outliers were detected in the dataset by applying a two-sided Grubbs´test (α = 0.05)

Next, we opted to explore neurochemical alterations in the brain of *Tsc2*^*+/−*^ rats by determining DA levels in post-mortem brain tissue. Despite absent changes in amphetamine response after systemic administration, the dopaminergic pathway remains a promising target due to its strong connection to repetitive behavior formation and general movement modulation [53, 56, 57]. An important DA target is the cortico-striatal-thalamo-cortical (CSTC) pathway, a neuronal circuit that is crucial for movement selection and initiation [57–59] and is implicated in influencing stereotyped behavior such as repetitive self-grooming and digging in rodents [60, 61]. Thus, we analyzed neurotransmitter levels in the striatum (CPu) and prefrontal cortex (PFC), as both are key areas regulating and modulating CSTC activity and function and are linked to stereotyped behavior initiation [62–64]. Therefore, region-specific HPLC for dopamine (DA) and downstream DA metabolites 3,4-dihydroxyphenylacetic acid (DOPAC) and homovanillic acid (HVA) was performed. While no significant alteration of DA level was found in *Tsc2*^*+/−*^ rat CPu tissue (Fig. 4A), significantly increased levels of DA were present within the PFC of *Tsc2*^*+/−*^ animals compared to wt (Fig. 4B; *F*(1, 18) = 4.478, *p* = 0.0485). Similarly, no changes in DA turnover were observed in CPu tissue (Fig. 4C). In the PFC, a sex x genotype interaction revealed a significantly decreased DA/(DOPAC + HVA) ratio in male *Tsc2*^*+/−*^ rats compared to female *Tsc2*^*+/−*^ and wt rats (Fig. 4D; *F*(1, 18) = 8.5, *p* = 0.01), suggesting significantly increased DA turnover in male *Tsc2*^*+/−*^ animals. Overall, a significant effect of genotype was found, revealing an increased DA turnover rate in *Tsc2*^*+/−*^ Eker rats compared to wt (*F*(1, 18) = 15, *p* = 0.001).

While PFC DA levels did not correlate with social interaction, they highly correlated with lower-order repetitive behaviors (nestlet shredding (*r*_*s*_(19) = 0.551, *p* = 0.01) and grooming (*r*_*s*_(19) = 0.514, *p* = 0.02)), further suggesting a connection between PFC-specific dopamine metabolism and the development of lower-order stereotypies in the Eker rat model.

## Discussion

Restricted repetitive behavior is one of the core pathological features of ASD and presents with high phenotypic symptom heterogeneity. Animal models of ASD are expected to offer construct validity, e.g., in the form of associated genetic mutations, and recapitulate characteristic key manifestations. Here, using a series of behavioral tests accounting for a spectrum of stereotyped behavior, we sought to characterize lower-order and higher-order repetitive behavior of the *Tsc2*^*+/−*^ haploinsufficient Eker rat. Importantly, we show that *Tsc2*^*+/−*^ Eker rats demonstrate a moderate phenotype of ASD-like behavior in the repetitive behavior domain involving increased lower-order motor stereotypies but no higher-order repetitive behavior, which was accompanied by a PFC-specific increase in DA levels.

While the Eker rat strain has been postulated as a model for ASD and ASD-like behavior [29, 31, 35, 36], to our knowledge, this is the first study to evaluate the repetitive behavior pattern in *Tsc2*^*+/−*^ Eker rats. Since the *Tsc2*^*+/−*^ Eker strain offers high construct validity and was indicated to present with social deficits, we hypothesized that Eker rats additionally develop repetitive stereotypies that are typically exacerbated under stress. Reported deficits include reduced novel object recognition and environmental exploration in the open field paradigm, as well as decreased social exploration in the form of non-anogenital [35] and anogenital exploration [37]. Interestingly, Petrasek et al. (2021) [37] further reported alterations in ultrasonic vocalization in *Tsc2*^*+/−*^ pups, suggesting phases of impaired social communication which is consistent with ASD characteristics in humans. A study on hippocampal plasticity reported a significant decrease in synaptic modification capacity in the form of reduced LTP and LTD amounts [33]. While this finding implies an impact on cognitive function, *Tsc2*^*+/−*^ animals were found to display unimpaired learning abilities in a Morris water maze paradigm [35], suggesting that molecular and functional changes are subtle and do not elicit a major behavioral impact [32]. Other TSC rodent models have been more thoroughly assessed regarding ASD-like behavior. *Tsc1*^*+/−*^ mice showed deficits in social interaction and impaired hippocampal learning revealed through the Morris water maze task and a fear conditioning test [65]. In a mouse model with Purkinje cell-specific *Tsc2*^*+/−*^ heterozygous genotype, repetitive digging behavior (marble burying test) and decreased social exploration, but no alterations in learning and memory were detected [66]. Interestingly, ASD symptom manifestations overall seem to be highly strain-dependent, as several heterozygous TSC rodent models present with specific combinations of social deficits, cognitive impairment and repetitive behavior. However, while social interaction and learning are commonly assessed domains in TSC rodent models, repetitive behavior analyses remain less frequently reported.

ASD-related rodent studies have focused on fixated action patterns such as obsessive self-grooming, digging, and nestlet shredding to quantify lower-order repetitive behavior [38, 40, 59, 67]. Here, *Tsc2*^*+/−*^ Eker rats were found to present with lower-order stereotypies in the form of increased nestlet shredding and self-grooming behavior. Conversely, no excessive digging was revealed in the marble burying test or occurred spontaneously. Instead, Eker rats spent significantly less time digging than wt rats. This contradictory finding fits the highly heterogeneous behavioral profiles of both human patients and rodent ASD models [57]. Some do not manifest repetitive behavior at all [68], others show anomalies only in specific behavioral tests. *Shank3* knockout mice, as an established ASD model, manifest repetitive behavior in the form of increased grooming and nestlet shredding but show decreased digging and marble-burying behavior [69–71]. Similar patterns can be found in *Shank2* mutant lines [57, 72]. Genetic factors exert great influence on innate digging behavior, thus, strain-variations in digging responses are common [40, 60]. Although excessive digging is commonly used as a measure for repetitive stereotypies, it can also be interpreted as an indicator of exploratory drive, depending on the motivational background [60]. Thus, reduced digging, especially in the habituation setting of this study, may correspond to a decrease in explorative behavior, which has been previously reported in *Tsc2*^*+/−*^ Eker rats [35].

Acute stress is known to play a role in altering repetitive behavior patterns, such as increasing grooming behavior [53, 73–75]. Thus, repetitive behavior was evaluated under stressful conditions in a newly established LSC test and the FST. The application of mild acute stress did exacerbate repetitive grooming behavior in *Tsc2*^*+/−*^ Eker rats, while increased stress abolished motor activity and promoted freezing behavior in both genotypes. Further, we report the absence of higher-order repetitive abnormalities in *Tsc2*^*+/−*^ Eker rats. Higher-order repetitive behaviors are often modeled using reversal learning paradigms such as the water T-maze or Morris water maze test [35, 47]. Animals are trained to prefer correct-rewarded over incorrect-unrewarded choices, after which the choice and reward relation is reversed. The number of trials needed to switch to the new correct-reward association is meant to reflect cognitive flexibility and insistence on sameness [11]. Together, Eker rats were found to present with mild changes in stereotypic behavior but seem to lack a higher-order repetitive behavior domain. Several ASD rodent models (C58 and BTBR T + tf/J mice) do not manifest with apparent cognitive inflexibility as they show unaltered responses in classical reversal learning paradigms. However, only when using a probabilistic reversal learning test approach with a reward rate of 80% instead of 100%, which is commonly used to model cognitive inflexibility in ASD patients, significant differences became apparent [76–78].

Importantly, we found that alterations in lower-order repetitive behavior in the Eker rat occur alongside previously reported social and cognitive behavioral patterns. Here, *Tsc2*^*+/−*^ animals showed mild impairments in the social recognition test, confirming deficits in the social domain of ASD manifestations [35, 37]. Interestingly, *Tsc2*^*+/−*^ rats maintained similar interaction time throughout all trials, while wt rats expectedly decreased the interaction time during the first four trials with the same partner and increased interaction time upon the introduction of a novel partner. Changes in the social recognition test can arise for several reasons, such as olfactory system impairments or locomotion deficits [79–81]. Additionally, decreased social interaction could be attributed to generalized alterations in exploratory drive that need to be specifically addressed. Potential cross-target effects should be excluded to increase the robustness of social deficit findings in *Tsc2*^*+/−*^ Eker rats. The social recognition test is thought to reflect both social interest and short-term recognition memory in rodent studies [79]. Thus, our results can be interpreted as both impaired social interest and target discrimination in Eker rats, as well as altered short-term recognition memory, suggesting an impact on cognitive function. Here, we show that the Eker model does not present significant changes in spatial learning and memory, as assessed in the WTM test. Our results fit previous reports showing typical learning and spatial memory in *Tsc2*^*+/−*^ animals in the Morris water maze, radial maze, and conditioned taste aversion test [35, 82]. On the contrary, *Tsc2*^*+/−*^ animals surprisingly show increased performance compared to wt upon modification to a delayed matching-to-place task meant to model episodic-like memory by adding a 2 h interval between trials [82]. However, improved performance in an episodic memory task can also be interpreted as a cognitive disadvantage; that is, Eker rats might perform better in finding novel platform locations due to a lack of memory formation of previous platform locations [82]. The entirety of observed brain malformations, cellular aberrations, and impairments of neuroplasticity suggests cognitive deficits to be present in the *Tsc2*^*+/−*^ Eker rat [21]. However, behavioral assessments so far have failed to show robust impairments in cognition. As discussed previously, applying higher task difficulty in the form of a probabilistic reversal learning test might be more sufficient to reveal higher-order repetitive behavior and cognitive inflexibility.

Given the growing understanding of sex discrepancies in ASD symptom manifestation [83, 84], sex effects are important to consider in ASD models. Thus, repetitive behavior alterations were assessed in both male and female *Tsc2*^*+/−*^ Eker rats. While little is known about sex-specific differences in ASD models, available data remains inconsistent with variance between animal strains and specific tasks [85, 86]. For example, the BTBR ASD model was found to present with repetitive grooming and marble burying in male but not female mice [87], while repetitive grooming in Nlgn4 ASD mice appears in female animals exclusively [88]. Here, we report a significant effect of sex before stress application in the LSC test, with increased digging behavior in female compared to male animals. Additionally, *Tsc2*^*+/−*^ females were found to show increased grooming behavior compared to both wt and males. In a *Tsc2*^*+/−*^ mouse model, female animals exhibited deficits in habituation response and following decreases in anxiety [89], suggesting that observed differences in repetitive behavior during the LSC test may arise from an impaired habituation response to the novel environment in female *Tsc2*^*+/−*^ rats. Further, *Tsc2*^*+/−*^ males presented with improved performance in the acquisition phase of the WTM. In line with our finding, *Tsc2*^*+/−*^ males are reported to show increased performance in an episodic memory task [82], suggesting altered memory formation in the Eker rat. Interestingly, this phenotype does not seem to occur in female Eker rats, suggesting sex differences in cognition; however, a confirmation and extension to other cognitive domains needs to be made before drawing meaningful conclusions. The here reported sex effects should be interpreted carefully, as male and female animal numbers are too small to draw meaningful conclusions. Overall, the discussed sex effects are considered to be of small relevance regarding the main concern of genotype effects in the Eker ASD model.

While the *Tsc2*^*+/−*^ Eker model presents with both social impairment and repetitive behavior, symptom expression remains mild. Deviations from human pathology in the *Tsc2*^*+/−*^ rat model have been proposed to originate from its genetic basis. Homozygous deletion is embryonically lethal in both mice and rats, thus, heterozygous TSC models are used [90, 91]. However, heterozygous animal models do not always accurately mirror typical neuropathy. In case of TSC, spontaneous seizure development as a hallmark manifestation cannot be recapitulated in animal models so far [21]. Often, “second hit paradigms” are therefore applied to model neurodevelopmental diseases with complex gene-environmental etiology, as they are thought to recapitulate human disease development more closely [21] and have been reported to induce ASD-like phenotypes [92]. Chemical “second hit” induction has been used to generate epileptic seizures in the *Tsc2*^*+/−*^ rat model and was found to increase ASD-associated behavior in mutant animals [35, 37]. Thus, introducing a “second hit” paradigm may exacerbate behavioral alteration in the Eker rat model, also with respect to its repetitive behavior profile. Finally, pharmacological testing of mTOR inhibitors or dopamine receptor antagonists and their effect on repetitive phenotypes, as well as lifespan analysis of stereotypies, especially considering early symptom onset in ASD, may be applied in the Eker model to challenge its translational accuracy and support predictive validity.

Various brain networks and dopamine-dependent signaling pathways have been linked to repetitive behavior formation [56, 57]. Here, we report alterations in PFC but not striatal DA levels of *Tsc2*^*+/−*^ animals, both of which are components of the CSTC pathway that regulates motor activity [57–59, 93]. The PFC has been shown to mediate repetitive behavior patterns [94, 95], is thought to be involved in goal-directed reinforcement learning [78, 96], and shows disrupted function in various animal models of ASD-linked risk genes and ASD patients [57]. Projections from the PFC into the substantia nigra pars compacta regulate the majority of DA release in the striatum. Striatal overactivation is further implicated in repetitive behavior development [64]. The *Scn1a*^*+/−*^ ASD mouse model, for example, exhibits hyperactivity and excessive self-grooming upon increased excitation in the PFC [97]. Observed increases in PFC DA levels that correlate with increased lower-order repetitive behavior thus strengthen our hypothesis of dopaminergic pathways being involved in the repetitive pathology found in *Tsc2*^*+/−*^ Eker rats. Interestingly, DA/(DOPAC/HVA) rates showed differences between sexes in *Tsc2*^*+/−*^ animals, suggesting that PFC-dependent DA increase causes compensatory changes through elevation of DA turnover in male *Tsc2*^*+/−*^ rats. While observed alterations in PFC-DA turnover may partially explain sex differences in repetitive behavior, extended mechanistic analysis of DA receptor and enzyme function are necessary to build this hypothesis and connect repetitive phenotypes to dysfunction of the dopaminergic PFC system. Considering that we did not find changes in homeostatic CPu DA levels, consistent with the absence of anhedonia, both regions possibly contribute differently to specific forms of repetitive behavior, with the striatum being involved in maintaining and flexibly changing choice patterns, thereby influencing reversal learning and behavioral flexibility [98]. We do recognize that other brain regions have been implicated in repetitive behavior formation, including the hippocampus, amygdala, and ventral tegmental area [57], which may in the future be addressed to characterize region-specific alterations of DA in the Eker rat. Additionally, the usage of animals for HPLC analysis that have undergone behavioral assessment to reduce animal numbers and comply with ethical standards raises the possibility of interference. To minimize this, a single low dose of amphetamine, established to not generate behavioral stereotypies in wt animals, was used for behavioral testing, and tissue was sampled after a one-day rest, circumventing acute DA alterations as well as long-term effects which manifest after 1–3 weeks [99] or after repeated treatment [100]. Lastly, alterations in dopaminergic signaling in the PFC are further known to result in changed social behavior and social recognition in ASD-associated animal models [101, 102]. This study lacks experimental evidence to causaly connect alterations in DA level to behavioral alterations; however, we demonstrate that changes in PFC-dependent DA levels occur simultaneously with ASD-like alterations in the social and repetitive behavior domain in *Tsc2*^*+/−*^ Eker rats.

## Conclusion

For the first time, we characterized the repetitive behavior profile of the *Tsc2*^*+/−*^ Eker rat model. In summary, we report the presence of lower-order repetitive behaviors that occurred alongside decreased social interaction in *Tsc2*^*+/−*^ Eker rats. Importantly, we showed that DA homeostasis is altered in the PFC of *Tsc2*^*+/−*^ rats, suggesting a contribution to ASD-like repetitive and social behavior manifestation. We propose that our findings add translational value to the usage of the *Tsc2*^*+/−*^ Eker rat model in preclinical ASD research.

## Data Availability

All data is provided within the manuscript.

## Abbreviations

ASD: Autism spectrum disorder
CPu: Striatum (caudate putamen)
CSTC: Cortico-striatal-thalamo-cortical
DA: Dopamine
DOPAC: 3,4-dihydroxyphenylacetic acid
FST: Forced swim test
HVA: Homovanillic acid
LSC: Light-sound-confinement test
LTD: Long-term depression
LTP: Long-term potentiation
mTORC1: Mammalian target of rapamycin complex 1
PFC: Prefrontal cortex
TSC: Tuberous sclerosis complex
wt: Wild-type
WTM: Water-T-maze
